# Supplementary material for: Avoiding 3D Obstacles in Mixed Reality: Does It Differ from Negotiating Real Obstacles?
Source: Sensors (Basel). 2020 Feb 17;20(4):1095. doi: 10.3390/s20041095 (PMC7071133; doi:10.3390/s20041095)
Supplement: Supplementary file 1 [file sensors-20-01095-s001.zip › Questionnaire S1.docx]

**Supplementary questionnaire S1**

1. ***User comfort***

1 = strongly agree, 2 = agree, 3 = neutral, 4 = disagree, 5 = strongly disagree

|  | **Participants** | **1** | **2** | **3** | **4** | **5** | **6** | **7** | **8** | **9** | **10** | **11** | **12** | **Mean** |
| --- | --- | --- | --- | --- | --- | --- | --- | --- | --- | --- | --- | --- | --- | --- |
| **1.1** | The HoloLens felt comfortable on my head during the experiment | 2 | 2 | 3 | 4 | 4 | 1 | 5 | 2 | 4 | 4 | 1 | 3 | 2.92 |
| **1.2** | The HoloLens was not properly mounted on my head when stepping over obstacles | 4 | 5 | 3 | 4 | 5 | 5 | 5 | 4 | 5 | 4 | 5 | 4 | 4.42 |
| **1.3** | The HoloLens stayed in place and did not shift | 2 | 1 | 4 | 3 | 3 | 1 | 1 | 2 | 1 | 2 | 1 | 1 | 1.83 |
| **1.4** | The HoloLens feels like normal glasses | 2 | 4 | 5 | 5 | 4 | 2 | 5 | 4 | 5 | 5 | 5 | 4 | 4.17 |
| **1.5** | The HoloLens blocked my view | 4 | 4 | 4 | 4 | 5 | 4 | 5 | 4 | 5 | 4 | 5 | 4 | 4.33 |
| **1.6** | I could normally look around with the HoloLens | 2 | 2 | 1 | 4 | 1 | 1 | 1 | 2 | 2 | 2 | 1 | 2 | 1.75 |
| **1.7** | The HoloLens was too heavy | 4 | 4 | 2 | 1 | 2 | 4 | 2 | 4 | 2 | 4 | 3 | 2 | 2.83 |
| **1.8** | I could walk around comfortably with the HoloLens | 2 | 1 | 4 | 4 | 3 | 1 | 5 | 2 | 2 | 2 | 1 | 3 | 2.50 |

1. ***Realism of the holographic obstacles***

1 = strongly agree, 2 = agree, 3 = neutral, 4 = disagree, 5 = strongly disagree

|  | **Participants** | **1** | **2** | **3** | **4** | **5** | **6** | **7** | **8** | **9** | **10** | **11** | **12** | **Mean** |
| --- | --- | --- | --- | --- | --- | --- | --- | --- | --- | --- | --- | --- | --- | --- |
| **2.1** | Brightness of holographic obstacles reduced obstacle-avoidance performance | 4 | 2 | 4 | 4 | 4 | 4 | 2 | 4 | 2 | 4 | 5 | 3 | 3.50 |
| **2.2** | Holographic obstacles resemble real ones | 2 | 4 | 3 | 2 | 2 | 1 | 2 | 3 | 5 | 2 | 4 | 2 | 2.67 |
| **2.3** | The texture of holographic obstacles reduced obstacle-avoidance performance | 4 | 4 | 4 | 4 | 5 | 4 | 2 | 4 | 4 | 4 | 5 | 4 | 4.00 |
| **2.4** | Holographic obstacles induce an avoidance response | 2 | 2 | 2 | 4 | 1 | 4 | 2 | 4 | 4 | 2 | 1 | 3 | 2.58 |
| **2.5** | The Field of View in which the holograms are visible reduced obstacle-avoidance performance | 4 | 4 | 4* | 3 | 3 | 4 | 2 | 3 | 1 | 2 | 5* | 3 | 3.17 |
| **2.6** | Holographic obstacles remained at the same position during measurements | 2 | 1 | 1 | 3 | 1 | 1 | 1 | 2 | 1 | 1 | 1 | 1 | 1.33 |
| **2.7** | Unnatural head movements were needed to clearly see the obstacle during the avoidance maneuver | 4 | 2 | 1 | 2 | 2 | 5 | 5 | 4 | 5 | 2 | 1 | 2 | 2.92 |
| **2.8** | Holographic obstacles seemed realistic | 2 | 3 | 2 | 2 | 1 | 1 | 2 | 3 | 4 | 2 | 3 | 2 | 2.25 |
| **2.9** | More attention was needed when avoiding real obstacles | 2 | 2 | 4 | 1 | 5 | 2 | 4 | 4 | 2 | 2 | 1 | 3 | 2.67 |
| **2.10** | The holographic obstacle blended well with the environment | 2 | 2 | 2 | 2 | 2 | 1 | 2 | 2 | 2 | 2 | 1 | 2 | 1.83 |
| **2.11** | I had to lift my foot higher to step over holographic obstacles than real ones | 2 | 2 | 1 | 1 | 5 | 3 | 1 | 4 | 2 | 2 | 5 | 3 | 2.58 |
| **2.12** | Avoiding holographic obstacles feels like avoiding real obstacles | 2 | 4 | 3 | 1 | 2 | 2 | 3 | 3 | 4 | 2 | 1 | 2 | 2.42 |
|  | | | | | | | | | | | | | | |
| **2.13** | How realistic are holographic obstacles on a scale from 1 to 10; 1 is not realistic, 10 is very realistic | 8 | 7 | 7 | 8 | 8 | 10 | 7 | 7 | 6 | 7 | 7 | 9 | 7.58 |

*These two participants probably confused the field of view in which holograms are presented with the overall field of view of the HoloLens itself, given the notable discrepancy with their general remarks (i.e., larger field of view for displaying holographic obstacles required) in Table 4 and Question 1.5 and 1.6 (overall field of view not blocked).

1. ***Usefulness of mixed-reality feedback***

*1 = strongly agree, 2 = agree, 3 = neutral, 4 = disagree, 5 = strongly disagree*

|  | **Participants** | **1** | **2** | **3** | **4** | **5** | **6** | **7** | **8** | **9** | **10** | **11** | **12** | **Mean** |
| --- | --- | --- | --- | --- | --- | --- | --- | --- | --- | --- | --- | --- | --- | --- |
| **3.1** | Feedback about obstacle avoidance performance was useful | 2 | 3 | 2 | 2 | 3 | 2 | 2 | 2 | 2 | 2 | 1 | 3 | 2.17 |
| **3.2** | By getting feedback, I made fewer mistakes while avoiding holographic obstacles | 2 | 4 | 2 | 4 | 4 | 4 | 1 | 2 | 1 | 2 | 2 | 4 | 2.67 |
| **3.3** | At the end of the experiment, I was better able to cross holographic obstacles than at the beginning | 2 | 2 | 1 | 3 | 2 | 4 | 1 | 2 | 1 | 2 | 1 | 3 | 2.00 |
|  | | | | | | | | | | | | | | |
| **3.4** | Which obstacle height was the hardest? | 0.4 | 0.4 | 0.4 | 0.4 | 0.4 | 0.4 | 0.4 | 0.4 | 0.4 | 0.4 | 0.4 | 0.4 |  |
|  | | | | | | | | | | | | | | |
| **3.5** | More attention was needed avoiding an obstacle after receiving feedback than before feedback | 4 | 2 | 2 | 4 | 4 | 3 | 2 | 3 | 4 | 2 | 1 | 3 | 2.83 |

1. ***General remarks***

| **Participants** | **Holographic obstacles improvements** | **MR feedback improvements** | **Other remarks** |
| --- | --- | --- | --- |
| **1** |  |  | It was an informative project, and I got a better impression of my mistakes with video feedback |
| **2** |  |  | The starting position relative to the bar was relatively short |
| **3** | Wider field of view HoloLens | Zoom out a little earlier, show more of the motion |  |
| **4** | The glasses were heavy and you had to look carefully to see the obstacles |  | Nice project to work on |
| **5** | Slightly more realistic (other colors) | Possibly indicate how good the avoidance maneuver was | Well done |
| **6** |  |  |  |
| **7** | Obstacles need to remain visible longer | Nothing, the feedback was good | Nice research |
| **8** |  |  |  |
| **9** | The colors of the obstacles need to be more realistic so they appear more robust. The visual image is very small so I quickly stopped seeing the obstacles | Maybe show more steps and also more tips besides just the visual feedback | I had the idea that black tape on the floor also helped me where the holographic image should be, while I could no longer see that image |
| **10** | Improving the view. In case you want to see the hologram, you are forced to look down with the HoloLens. If you look straight ahead, you will not see a hologram |  |  |
| **11** | That you continue to see the obstacles when you cross it. When they disappeared, I stepped through them more often instead of over |  | It was tiring, but nice to take part in such a study. Because of the variety it was good to keep going |
| **12** | The angle in which you can still see them may be larger | For example a value for the distance of the obstacle |  |
